# Relationships among Antibodies against Extractable Nuclear Antigens, Antinuclear Antibodies, and Autoimmune Diseases in a Brazilian Public Hospital

**DOI:** 10.1155/2018/9856910

**Published:** 2018-09-30

**Authors:** Fernanda Weyand Banhuk, Bruna Corrêa Pahim, Alex Sandro Jorge, Rafael Andrade Menolli

**Affiliations:** ^1^Laboratory of Applied Immunology, Center of Medical and Pharmaceutical Sciences, Western Parana State University, Cascavel/PR 85819-110, Brazil; ^2^Laboratory of Clinical Immunology, Western Parana University Hospital, Western Parana State University, Cascavel, PR 85806-470, Brazil

## Abstract

One characteristic of autoimmune diseases (ADs) is the production of autoantibodies for extractable nuclear autoantigens, which may aid in the discrimination of the different types of autoimmune diseases and is related to different antinuclear antibody (ANA) patterns. The present study verified the profile of patient samples tested for extractable nuclear antigens (ENA) antibodies in a public hospital and correlated the ENA results with ANA patterns and patient diagnoses. The study reviewed data in the medical records of patients who underwent anti-ENA tests at a public hospital in the West of the State of Paraná from February 2011 to January 2017. Patients were classified according to age, ethnicity, gender, anti-ENA test results, ANA results, and the presence or absence of AD. Thirty-six (20.9%) samples of the 172 anti-ENA tests were positive, seven (4.1%) samples were undetermined, and 129 (75%) exhibited negative results. The ANA reagent was found in 84.3% of the anti-ENA-positive samples. The anti-SSA/Ro autoantibody exhibited the highest frequency in the group, 41.7% (15/36). The most common pattern was nuclear fine speckled, which was found in 24.3% of the samples. The association results indicated a significant relationship between ANA titer and diagnosis in the anti-ENA- and ANA-positive patients. The anti-ENA-negative patients were diagnosed with an AD in 35% (45/129) of the cases, and 75% (27/36) of the anti-ENA-positive patients were diagnosed with an AD. Systemic lupus erythematosus and scleroderma were the most common pathologies in the antigen-positive patients. The anti-ENA test is a good marker to aid in the complex clinical diagnosis of patients with autoimmune diseases.

## 1. Introduction

Multiple factors cause autoimmune diseases and involve a wide variety of genes and environmental factors, such as stress, age, sex, hormones, and infection exposure [1]. Autoimmune diseases are characterized by autoaggression of the immune system against constitutive antigens of an individual via production of autoantibodies, which exhibit clinical significance when associated with other disease manifestations [2, 3].

The detection of antibodies against cellular antigens (AACA) in HEp-2 cells, also known as antinuclear antibodies (ANA HEp-2), using indirect immunofluorescence (IFI) is the methodology of choice for the screening and identification of various autoantibodies [4, 5]. The ANA assay detects a range of antibodies that react with antigens in the nucleus, nucleolus, cytoplasm, and mitotic cellular apparatus [6]. However, this test should be complemented by the research and identification of autoantibodies and specific autoantigens, many of which exhibit great clinical utility and may play roles as diagnostic markers, as prognostic indicators, or for the monitoring of autoimmune diseases [7–9]. In addition, the presence of positive ANA does not necessarily indicate a disease state, because low levels of ANA are detected in 30% of healthy individuals [10, 11].

The most frequent antigens described in autoimmune diseases exhibit a nuclear localization and are called extractable nuclear antigens because of the purification process; they are most commonly represented by the acronym ENA (*extractable nuclear antigens*) [12].

Anti-ENA research is used to identify a group of specific autoantibodies, including anti-SSA/Ro, anti-SSB/La, anti-RNP, anti-Sm, anti-Scl-70, anti-Jo-1, anti-CENP-B, anti-NUC, and anti-dsDNA. These autoantibodies are detected using several methodologies, such as immunoblot, counterimmunoelectrophoresis, immunodiffusion, enzyme-linked immunosorbent assay (ELISA), and hemagglutination. However, variations in the results can be found because these techniques differ in sensitivity and specificity [13–15]. DNAds as an antigen is not included as ENA by some authors, because the anti-DNA test is commonly performed with another methodology (IIF with Crithidia as antigen), but in this study it was named as ENA, once the sera of patients were tested together with the other ENA antigens by immunoblot method. It is important to note that although the Jo-1 antigen is common in the ENA group, it is a cytoplasmic antigen.

Further analysis of reactivity to ENA may contribute to an improved discrimination among the different types of autoimmune rheumatic diseases (ARD). For example, the presence of anti-ribonucleoprotein (RNP) antibodies is part of the diagnosis of mixed connective tissue disease (MCTD), and positive results for ANA and the presence of anti-dsDNA or anti-Sm constitute three of the six immunological criteria for the diagnosis of systemic lupus erythematosus (SLE) [16, 17]. The presence of antibodies directed against SS-A (Ro) or SS-B (La) ribonucleoproteins is a criterion for the diagnosis of Sjögren's syndrome (SS), and the appearance of antibodies against histidyl-sRNA synthetase (Jo-1) is a major immunological characteristic of polydermatomyositis. The appearance of anti-centromere antibodies (CENP-B) or topoisomerase 1 (Scl-70) aids in the diagnosis of systemic sclerosis [18–20].

The presence of anti-ENA is related to the different patterns of ANA tests, which are associated with manifestations of some autoimmune diseases. Therefore, the present study retrospectively evaluated the correlation between anti-ENA-positive and -negative sera, ANA patterns, and clinical data.

## 2. Material and Methods

### 2.1. Casuistry

A cross-sectional, uncontrolled study was performed via a review of the medical records of patients who underwent anti-ENA examination at the Western Paraná University Hospital (HUOP) from February 2011 to January 2017. Patients were selected only on the condition that they had undergone the anti-ENA test, i.e., not considering if preliminary tests were performed, such as the ANA test, since it is common to perform the anti-ENA test as a complementary test to the ANA test.

Patients were classified according to age, ethnicity, gender, anti-ENA, and ANA test results, diagnosis, and specialty that requested the tests. These factors were collected from records in the Tasy® electronic hospital management system, which allowed for verification of laboratory tests issued and stored and the test results in the support, diagnostic, and therapeutic service (SDTS), and patient's archive/history department. The averages and percentages were obtained from the data of each patient.

The ethics committee in research involving human beings of the Western Paraná State University approved the study under number 861.960.

### 2.2. ANA and Anti-ENA

The ANAs were determined semiquantitatively in HEp-2 cells using immunofluorescence (Viro-Immun Diagnostics GmbH), according to the manufacturer's protocol. The slides were read on an Olympus epifluorescence microscope, with a mercury vapor lamp of 100 watts power, by two observers, at 400X magnification. Samples were classified into reactives or non-reactives by comparing the fluorescence intensity observed for the sample and the fluorescence intensity observed in the control slide (FITC-QC slide Immuno Concepts N.A. Ltd.) at the second reactivity threshold level (1+). According to the staining of the nucleus and cytoplasm of the cells, different patterns were described, following the Brazilian consensus of ANA-HEp2.

Anti-ENA research was performed using a membrane-based immunoblot assay (Nucleo-9-Line, ORGENTEC Diagnostika GmbH) for the semiquantitative determination of IgG autoantibodies against dsDNA, nucleosome (NUC), SS-A (Ro), SS-B (La), Sm, RNP/Sm, Scl-70, Jo-1, and CENP-B antigens in serum or plasma.

### 2.3. Statistical Analysis

An association between the diagnosis of AD and ANA patterns, ANA titer, and diagnosis were tested for positive and negative anti-ENA patients using the nonparametric chi-square test with 95% reliability [21]. Patients with indeterminate anti-ENA results lacked sufficient data for conclusive statistical tests, and these data were excluded. The tests were run in software R version 3.4.1 [22] using FunChisq [21] and the ggplot2 graphic package [23].

## 3. Results

Thirty-six (20.9%) of the 172 anti-ENA tests performed between 2011 and 2017 were positive, seven (4.1%) samples were undetermined, and 129 (75%) exhibited a negative result. These patients showed an age range from 4 to 80 years, averaging 38.01 years, with a prevalence of females and white ethnicity (77.3% and 78%, respectively).

Table S1 (Supplementary Material) presents the behavior of patients with anti-ENA-positive sera with respect to ANA patterns and titers, diagnosis for autoimmune diseases, and sociodemographic data.

The anti-ENA-positive patients aged from 13 to 71 years, averaging 41.8 years, and a prevalence of females and white ethnicity was observed (86.1% and 77.7%, respectively).

ANA positivity in anti-ENA-positive patients was found in 84.3% (27/32) of the samples analyzed that underwent the ANA test. Five of the 36 anti-ENA-positive patients exhibited negative ANA, and the ANA test was not performed in four patients.

The anti-SSA/Ro autoantibody exhibited the highest frequency in the group (41.7%; 15/36). Eleven sera were exclusive for anti-SSA/Ro, and four sera were associated with other autoantibodies. The other most frequently isolated autoantibodies were anti-NUC (13.9%), followed by anti-Scl-70 (11.1%), anti-CENP-B and anti-RNP/Sm (both 8.33%), and SS-B (La) (5.6%). Four of the five patients who were anti-ENA-positive and ANA-negative were anti-SS-A/Ro-positive patients.

The seven patients with undetermined results for anti-ENA included 42.8% who were indeterminate for SS-A (Ro), and four ANA-positive and three ANA-negative patients.

The following ANA patterns occurred in patients with positive anti-ENA (patients in whom it was possible to obtain such data): nuclear fine speckled (28.1%); nuclear large/coarse speckled (12.5%); nuclear dense fine speckled (9.4%); centromere (8.3%); nuclear homogeneous (6.3%); and nucleolar pattern (5.6%). The presence of more than one ANA pattern was verified in three samples, which were identified as a mixed pattern (8.3%).

Some anti-ENA-positive patients (27.8%) did not exhibit ANA patterns or titers because the test was negative or unsolicited.

The association results indicated a significant relationship between ANA titer and diagnosis in anti-ENA- and ANA-positive patients (*p value*=0.0069) (Table 1).

Most anti-ENA-negative patients were women (73.6%), white (79.7%), and aged from four to 80 years (average of 36.3 years). Positive ANA results were observed in 47.3% of the samples (54/112) (no results for the ANA test were found in the medical records of 17 anti-ENA-negative patients). The following immunofluorescence patterns were observed: nuclear fine speckled pattern (37.7%); nuclear dense fine speckled (18.9%); mixed patterns (18.9%); nuclear large/coarse speckled (9.4%); nuclear homogeneous (7.8%); cytoplasmic (7.5%); nucleolar (1.9%); and mitotic apparatus (1.9%).

Complete information about the immunofluorescence pattern was not obtained in some anti-ENA-negative/ANA-positive samples. Only the titer was recorded in these samples.

Table 2 provides that most (65.4%) anti-ENA-negative patients exhibited an intermediate titer range (positive/negative diagnosis). However, Table 1 shows that 76.9% of the samples were obtained from anti-ENA-positive patients in the highest range. We also observed that 84.6% of anti-ENA- and ANA-positive patients had a diagnosis of AD (Table 1), and only 71.1% of the anti-ENA-negative and ANA-positive patients had an AD diagnosis.

Anti-ENA-negative and ANA-positive patients exhibited no association between ANA and AD diagnosis (*p value=* 0.2983). Two patients were not included in Table 2 because no data of titers in their ANA tests was found.

There were no significant associations between the different ANA patterns and AD diagnoses in positive and negative anti-ENA patients, suggesting no specific association of ANA patterns with AD for these samples.

Of the total number of anti-ENA tests performed, 14 (8.1%) were requested by rheumatologists and 158 (91.9%) by physicians from other specialties. At the anti-ENA-positive tests, 16.7% were requested by rheumatologists and the others by other specialties, while in anti-ENA negative tests, rheumatologists were responsible for the request of only 6.2% of the exams.

Twenty-seven (75%) of the 36 anti-ENA-positive patients were diagnosed with an AD. Nine of these patients (33.3%) were diagnosed with SLE, and five patients were diagnosed with SLE associated with another autoimmune disease (Table 3). Anti-ENA-negative patients were diagnosed with an autoimmune disease in 35% of the sample (45/129). Twelve (26.7%) of these patients were diagnosed with SLE, and four (8.9%) patients were diagnosed with SLE associated with another autoimmune disease.

## 4. Discussion

Numerous studies have demonstrated that a positive ANA test is a strong indicator of an autoimmune disease, and this test is a good methodology to extensively screen for autoimmunity. However, progressive and vigorous improvements in the technology of the various elements composing the assay, including the quality of the HEp-2 cell slides, fluorescent conjugates, and fluorescence microscopes, have revised this concept [24].

These technological improvements greatly increased test sensitivity, and current tests detect antibodies at lower serum levels and less avidity than earlier assays. Therefore, the screening for antibodies against cellular antigens also exhibits a lower specificity [25]. In addition, the prescription of the ANA test started to be made by a broad spectrum of medical specialists, which was once primarily prescribed solely by rheumatologists. Therefore, the pretest probability of autoimmunity was high and favored the diagnostic performance of the test [24, 26].

A wide variety of specialists, who obviously treat different patients in whom the diagnosis of autoimmune rheumatic disease is less prevalent, are requesting ANA examinations with less discretion. Therefore, the chance for positive results in healthy individuals or individuals with less expressive clinical presentations is greater [27–29]. This increase shows the importance of requesting tests for the identification of specific autoantibodies after receiving a positive ANA test.

Our results demonstrated that 76.9% of the anti-ENA-positive patients were in the highest ANA titer range, and 84.6% of patients who were anti-ENA- and ANA-positive were diagnosed with an AD. This result is very similar to that reported by Jeong [30], who used an anti-ENA test with the same methodology (line immunoassay) and found 83.9% positivity in ANA and anti-ENA tests in a cohort of Asian patients with AD. A study in Bangladesh [31] showed autoimmune diseases in 85.5% of double positivity (ENA and ANA tests), working with dot-blot methodology for ENA detection. This demonstrates that association of these two tests (ANA for screening and anti-ENA to confirm) is essential for diagnosis of AD [10].

Anti-ENA-positive with ANA-negative was found in five (15.2%) sera, which was unexpected because ANA-Hep2 tends to have a higher sensitivity than immunoblot tests. However, the occurrence is not uncommon [12, 27, 32] and some of these ANA-negative results could be patients in immunosuppressive therapy [33]; a revision of their medical records should be necessary to clarify this point. Although five samples showed ANA-negative with anti-ENA-negative, only one patient was diagnosed with AD. The methodology used in this objective is important, as Kidd [34] argued that samples that were previously negative turned out to be positive when subjected to another analysis with a different methodology. Finally, the association of different techniques ensures greater sensitivity and specificity [30].

Anti-SSA/Ro are the antigens that cause the higher number of “false-positive” anti-ENA tests [35]. In this study, this happened in four of five ANA-negative results with anti-ENA-positive. This gives rise to the question that it was highly recommended to use anti-SSA antibody assays in addition to the ANAHEp-2 test in the function of this characteristic [36]. Based on these observations, over three decades ago a transfected HEp-2 cell line overexpressing SS-A/Ro60 was developed and this cell-based IIF assay was marketed as HEp- 2000 cells [37]. According to Bossuyt et al. [38], this characteristic of SSA/Ro happened due to the loss of this antigen in the process of fixing HEp-2 cells to make ANA test kits.

Another point is the sensibility of different methodologies or different antigens used in ENA tests, with authors demonstrating that in some laboratories line immunoassays have also been used as a screening test for disease-specific autoantibodies that are seen in SLE, Sjögren's syndrome, Idiopathic Inflammatory Myopathies, paraneoplastic, and autoimmune liver diseases [35]. Also, the methodology of LIA used here shows good sensitivity, and various studies demonstrate a great correlation between data obtained by LIA and other techniques. Chandratilleke et al. [12] compared LIA with an automatized fluorescent assay (FIDIS) in 529 samples and found a 90.5% concordance. A work conducted in Korea [30], with patients with AD, showed a similar performance among three anti-ENA methods (two automatized immunoenzymatic assays and LIA). Vercammen et al. [39] analyzed 174 ANA-positive samples by immunodiffusion, LIA, and an automatized fluorescent immunodetection methodology, showing no difference between them. However, most studies with comparisons between different techniques of anti-ENA tests are conducted with patients who have been diagnosed with AD.

The highest frequency of the anti-SSA/Ro autoantibody (43.2%) in anti-ENA-positive patients was also reported by Lora et al. [40], with 67.7% predominance, and Sanchez-Guerrero et al. [41], who reported a 40% frequency of antigen-positive samples.

The most prevalent association of autoantibodies in the present study was anti-RNP/Sm (27%), which is similar to the results of Arbuckle et al. [42], who found a prevalence of anti-RNP/Sm in 32% of the samples analyzed. The ANA-HEp2 pattern associated with RNP and Sm autoantigens was nuclear large/coarse speckled [43]. Four of the five patients who exhibited the nuclear large/coarse speckled pattern, although this was associated with mixed patterns of ANA-HEp2, also exhibited anti-RNP/Sm in their sera and a clinical association with SLE.

In general, patients with autoimmune diseases tend to exhibit moderate (1/160 and 1/320) and high (≥ 1/640) ANA titers, and healthy subjects with positive ANA tend to exhibit low titers (1/80) [15, 44]. However, exceptions may exist in both cases [45]. In this study, a significant association was detected between ANA titers and the presence of ADs in patients who were anti-ENA-positive; and these patients exhibited the highest ANA titers (≥1: 640), which is consistent with the literature [15, 25]. Craig [46] showed that, in a large cohort, the number of diagnoses of AD increased with the titer of ANA (13.3% in low levels to 43.4% in high levels), which is consistent with the present data; diagnosis of AD occurred in 55 (71.14%) patients with high titers of ANA (>1:160). Thus, the chance of finding anti-ENA increases with increasing ANA titers [28, 46], as demonstrated in this study, with only one sample with anti-ENA-positive results in the range of lower titers of ANA-positive results.

The ANA patterns for anti-ENA-positive and -negative sera were not significantly associated with ADs. Therefore, it was not possible to establish a relationship between an ANA pattern and a specific disease in this sample.

The homogeneous nuclear fluorescence pattern is primarily associated with systemic autoimmunity [47]. This pattern was observed in two patients who had a clinical condition of SLE: one of these patients exhibited the anti-SSA/Ro antibody; the other patient exhibited anti-Scl-70, anti-Smith, and anti-RNP/Sm; and the last two antibodies listed are markers for SLE [48]. Three of the five patients diagnosed with scleroderma exhibited anti-CENP-B and a centromere pattern (NC) in the ANA test. Göring et al. [49] reported a strong association between scleroderma, anti-CENP-B, and NC pattern.

The NDFS pattern is frequently observed in people without autoimmune diseases and with a positive ANA test [44]. However, the present study observed that two of the three patients who exhibited the nuclear dense fine speckled pattern were diagnosed with rheumatic autoimmune diseases. This is corroborated by some authors [50, 51], who showed that the antigen most related with NDFS pattern is DSF70, but the appearance of others antigens can occur when the sample is from a patient with an autoimmune disease. The frequency of NDFS in other studies varied from 0.3 to 27% [50, 52, 53], showing that differentiation between the NDFS pattern and other patterns can be difficult and it is likely that laboratories in the past have reported NDFS as a mixed pattern. IIF is a subjective test and recognition of this pattern is open to interpretation [54].

A high prevalence of rheumatic autoimmune diseases (75%) was found in anti-ENA-positive patients. The most common diagnosis in these patients was SLE (33.3%), which is consistent with Lora et al. [40], who evaluated the ANA patterns and clinical diagnoses of anti-SS-A/Ro positive patients, and SLE was also the predominant clinical association in these patients with 50.8% frequency.

The diagnostic accuracy of anti-ENA antibodies in AD patients is known to vary with patient selection and detection technique. Previous studies conducted by Albon et al. [55] and Vercammen et al. [39] have compared several methods of ENA testing and shown overall equivalence for most antigens. However, unlike the approach of this study, recruitment in these studies and others [30, 31, 56] was based on patients with known diseases or a positive ANA, which alters the pretest probability of a positive ENA test.

Some associations between extractable nuclear antigens and AD are close and frequently reported in the literature, for example, between Scleroderma and Scl-70 or Sjögren's Syndrome and SS/A or SS/B [2, 3], which were found in this study. Nevertheless, some uncommon associations occurred between anti-ENA and clinical manifestations, as rheumatoid arthritis (RA) and SS/A or autoimmune hemolytic anemia and nucleosome. Association between antibodies against ENA and RA occurred in a minority of patients and was not related to symptoms, and a variety of antigens could occur in RA [57, 58]. Occurrence of anti-ENA in patients with Behçet's disease is uncommon, around 10% [59, 60], but the most frequent antigen is SS/A. An association found in this work, which has not been reported in the literature, was between autoimmune hemolytic anemia and anti-nucleosome. Hemolytic anemia is a pure autoimmune disease and usually does not entail the presence of autoantibodies such as ANA or anti-ENA. In this case, it should be an initial manifestation of some systemic autoimmune diseases, such as SLE [17].

Forty-five of the anti-ENA-negative patients (35%) exhibited manifestations of AD. Twelve of these patients (26.7%) were diagnosed with SLE, and four patients were diagnosed with SLE associated with other autoimmune diseases. These results show the importance of the clinical association of the ANA examination, which is the screening test for autoimmunity [15].

Seven of the 172 antigen tests performed exhibited antibody levels that were very close to the detection limit, and these patients had inconclusive or undetermined anti-ENA results. Two of these patients exhibited clinical signs of AD, and no information on the diagnosis was found for two other patients.

The sensitivity of the tests performed, the experience of the professionals during the execution, interpretation of the results, erroneous requests for anti-ENA tests, and the lack of ANA results when necessary are the primary limitations of the present study. ANA examination should be requested only in the presence of a convincing suspicion of autoimmune disease because a positive result does not necessarily imply autoimmunity. However, when a positive ANA result occurs, it is important to identify the specific autoantibody involved.

## 5. Conclusions

The anti-ENA test was a good method to aid in the clinical diagnosis of rheumatological autoimmune diseases. In this study, several anti-ENA tests have been solicited without an ANA test, which should not occur once sensitivity of the first test is lower than the second test.

It is essential to characterize the presence of antibodies that are particular to autoimmune pathologies using specific techniques in ANA-positive patients.

Professionals that directly or indirectly work with anti-ENA and ANA examinations must perform an accurate and constant review of the paradigms that guide the interpretation of the results so that the clinical diagnosis and subsequent treatment of the patients can be successfully achieved.

## Figures and Tables

**Table 1:** Titers of ANA tests and clinical diagnosis of autoimmune diseases in anti-ENA-positive patients.

ANA Titles	Diagnosis AD	
	N	Y^*∗*^
1:80 – 1:160	0	1
1:160- 1:320	3	2
1:640 - >1:640	1	19

Y = presence of autoimmune disease; N = no diagnosis of autoimmune disease; *∗*  =  positive association between AD presence and ANA titers.

**Table 2:** Titers of ANA tests and clinical diagnosis of autoimmune diseases in anti-ENA-negative patients.

ANA Titles	AD Diagnosis	
	N	Y
1:80 – 1:160	3	3
1:160- 1:320	10	24
1:640 - >1:640	2	10

Y = presence of autoimmune disease; N = no diagnosis of autoimmune disease.

**Table 3:** Distribution of autoimmune diseases and autoantibodies against extractable nuclear antigens (anti-ENA).

AD in Anti-ENA-positive patients	Antigens found
SLE (9/27)	SS-A (Ro), SS-B (La), Smith, Scl-70, NUC, dsDNA, RNP/Smith
Scleroderma (4/27)	SS-A (Ro), Scl-70, CENP-B
Sjögren's syndrome (3/27)	SS-A (Ro), SS-B (La)
Polymyositis (2/27)	SS-A (Ro), Smith, Scl-70, RNP/Smith
Behçet's disease (1/27)	SS-A (Ro)
Rheumatoid arthritis (1/27)	SS-A (Ro), SS-B (La), Smith, Scl-70, CENP-B, Jo-1, RNP/Smith
Autoimmune hemolytic anemia (1/27)	NUC
Autoimmune hepatitis and scleroderma (1/27)	CENP-B
SLE and scleroderma (1/27)	Scl-70
SLE and polymyositis (1/27)	RNP/Smith
SLE, MCTD and scleroderma (1/27)	RNP/Smith
SLE and Evans syndrome (1/27)	NUC
SLE and Sjögren's syndrome (1/27)	SS-A (Ro), Smith, RNP/Smith

SLE = systemic lupus erythematosus.

MCTD = mixed connective tissue disease.

## Data Availability

The data used to support the findings of this study are included within the article.

## Supplementary Materials

Supplementary Materials Table S1: sociodemographic data, patterns, and titers of ANA and diagnosis of anti-ENA-positive patients.
