# Supplementary material for: Relationships among Antibodies against Extractable Nuclear Antigens, Antinuclear Antibodies, and Autoimmune Diseases in a Brazilian Public Hospital
Source: Autoimmune Dis. 2018 Sep 30;2018:9856910. doi: 10.1155/2018/9856910 (PMC6186355; doi:10.1155/2018/9856910)
Supplement: Supplementary Materials — Table S1: sociodemographic data, patterns, and titers of ANA and diagnosis of anti-ENA-positive patients. [file 9856910.f1.docx]

Table S1 - Sociodemographic Data, Patterns and Titers of ANA and Diagnosis of Anti-ENA-Positives Patients

| Gender | Age | Ethnicity | Panel ENA | ANA | ANA Patterns | ANA Titers | Diagnosis AD |
| --- | --- | --- | --- | --- | --- | --- | --- |
| F | 55 | White | SS-A (Ro) | P | Hom | (1:640 - >1:640) | Y |
| F | 50 | White | SS-A (Ro) | UR | UR | UR | Y |
| F | 22 | White | SS-A (Ro) | P | NDFS | (1:640 - >1:640) | Y |
| M | 42 | White | Scl-70 | P | NDFS | (1:160 - 1:320) | N |
| F | 44 | Black | SS-A (Ro); Smith; RNP/Sm | P | NLCS | (1:640 - >1:640) | Y |
| F | 45 | White | RNP/Sm | P | NLCS | (1:640 - >1:640) | Y |
| F | 27 | White | Smith; RNP/Sm | P | Mixed | (1:640 - >1:640) | Y |
| F | 43 | White | SS-B (La) | P | NFS | (1:640 - >1:640) | Y |
| F | 51 | White | SS-A (Ro) | P | Nucleolar | (1:640 - >1:640) | Y |
| M | 59 | White | SS-A (Ro) | N | N | N | N |
| F | 39 | White | SS-A (Ro) | N | N | N | N |
| M | 31 | White | RNP/Sm | UR | UR | UR | Y |
| F | 71 | White | CENP-B | P | NC | (1:640 - >1:640) | Y |
| F | 49 | White | SS-B (La) | P | Mixed | (1:640 - >1:640) | Y |
| F | 71 | White | SS-A (Ro) | UR | UR | UR | N |
| F | 20 | White | NUC | P | NFS | (1:640 - >1:640) | Y |
| F | 19 | White | Smith; RNP/Sm | P | NFS | (1:640 - >1:640) | Y |
| F | 52 | Brown | CENP-B | P | NC | (1:640 - >1:640) | Y |
| F | 51 | White | SS-A (Ro) | P | NFS | (1:640 - >1:640) | Y |
| M | 19 | White | Smith; Scl-70; RNP/Sm | P | Hom | (1:640 - >1:640) | Y |
| F | 50 | Brown | CENP-B | P | NC | (1:640 - >1:640) | Y |
| F | 48 | NI | SS-A (Ro); SS-B (La); Smith; Scl-70; CENP-B; Jo-1; RNP/Sm | P | NFS | (1:40 - 1:80) | Y |
| F | 32 | White | NUC | N | N | N | N |
| F | 62 | White | NUC | P | Mixed | (1:160 - 1:320) | N |
| F | 47 | Brown | SS-A (Ro); SS-B (La) | P | NFS | (1:640 - >1:640) | Y |
| F | 29 | White | NUC | P | NFS | (1:640 - >1:640) | N |
| F | 29 | White | SS-A (Ro) | P | Nucleolar | (1:160 - 1:320) | N |
| F | 42 | Black | Scl-70 | P | NDFS | (1:160 - 1:320) | Y |
| F | 42 | Brown | SS-A (Ro) | N | N | N | Y |
| F | 55 | White | Scl-70 | N | N | N | N |
| F | 69 | White | Scl-70 | P | NFS | (1:640 - >1:640) | Y |
| F | 17 | White | NUC; dsDNA | P | NFS | (1:160 - 1:320) | Y |
| F | 52 | White | SS-A (Ro); NUC; dsDNA; RNP/Sm | P | NLCS | (1:640 - >1:640) | Y |
| M | 13 | NI | RNP/Sm | P | * | * | Y |
| F | 27 | White | NUC | P | NLCS | (1:640 - >1:640) | Y |
| F | 32 | White | SS-A (Ro) | UR | UR | UR | Y |

F = female; M = male; NI = no information; UR = non-perfomed; P = positive; N = negative; Y = presence of autoimmune disease; N = there was no diagnosis of autoimmune disease; Hom = homogeneous nuclear; NFS = nuclear fine speckled; NDFS = nuclear dense fine speckled; NLCS = nuclear large/coarse speckled; NC = centromere; Mixed = identification of more than one ANA fluorescence pattern. AD = autoimmune diseases. * = it was not possible to obtain data from the patient's chart.
